# Establishing research priorities in prevention and control of vector-borne diseases in urban areas: a collaborative process

**DOI:** 10.1186/s40249-018-0463-y

**Published:** 2018-09-03

**Authors:** Christian Dagenais, Stéphanie Degroote, Mariam Otmani Del Barrio, Clara Bermudez-Tamayo, Valéry Ridde

**Affiliations:** 1 Department of Psychology, University of Montreal, Pavillon Marie-Victorin, Bureau C-355, C. P. 6128, succursale Centre-ville., Montreal, QC H3C 3J7 Canada; 2 University of Montreal Public Health Research Institute, Montreal, QC Canada; 3 Vector, Environment and Society Unit, Special Programme for Research and Training in Tropical Diseases (TDR), World Health Organization, Geneva, Switzerland; 4 Andalusian School of Public Health, Granada, Spain; 5 CIBER of Epidemiology and Public Health (CIBERESP), Madrid, Spain; 6 IRD (French Institute for Research on Sustainable Development), CEPED (IRD-Université Paris Descartes), Universités Paris Sorbonne Cités, ERL INSERM SAGESUD, Paris, France

**Keywords:** Concept mapping, Research priorities, Vector-borne diseases, Urban areas

## Abstract

**Background:**

In 2015, following a call for proposals from the Special Programme for Research and Training in Tropical Diseases (TDR), six scoping reviews on the prevention and control of vector-borne diseases in urban areas were conducted. Those reviews provided a clear picture of the available knowledge and highlighted knowledge gaps, as well as needs and opportunities for future research. Based on the research findings of the scoping reviews, a concept mapping exercise was undertaken to produce a list of priority research needs to be addressed.

**Methods:**

Members of the six research teams responsible for the “VEctor boRne DiseAses Scoping reviews” (VERDAS) consortium’s scoping reviews met for 2 days with decision-makers from Colombia, Brazil, Peru, Pan-American Health Organization, and World Health Organization. A total of 11 researchers and seven decision-makers (from ministries of health, city and regional vector control departments, and vector control programs) completed the concept mapping, answering the question: “In view of the knowledge synthesis and your own expertise, what do we still need to know about vector-borne diseases and other infectious diseases of poverty in urban areas?” Participants rated each statement on two scales from 1 to 5, one relative to ‘priority’ and the other to ‘policy relevance’, and grouped statements into clusters based on their own individual criteria and expertise.

**Results:**

The final map consisted of 12 clusters. Participants considered those entitled “Equity”, “Technology”, and “Surveillance” to have the highest priority. The cluster considered the most important concerns equity issues, confirming that these issues are rarely addressed in research on vector-borne diseases. On the other hand, the “Population mobility” and “Collaboration” clusters were considered to be the lowest priority but remained identified by participants as research priorities. The average policy relevance scores for each of the 12 clusters were roughly the same as the priority scores for all clusters. Some issues were not addressed during the brain-storming. This is the case for governance and for access and quality of care.

**Conclusions:**

Based on this work, and adopting a participatory approach, the concept mapping exercise conducted collaboratively with researchers from these teams and high-level decision-makers identified research themes for which studies should be carried out as a priority.

**Electronic supplementary material:**

The online version of this article (10.1186/s40249-018-0463-y) contains supplementary material, which is available to authorized users.

## Multilingual abstracts

Please see Additional file 1 for translations of the abstract into the six official working languages of the United Nations.

## Background

The rapid growth of cities in low- and middle-income countries is altering disease patterns and vector dynamics and increasing the risk of transmission of infectious diseases, including vector-borne diseases (VBDs) [1, 2]. In a rapidly changing global scenario, the recent Resolution on Global Vector Control Response 2017–2030 adopted by World Health Organization (WHO) Member States in June 2017 at the World Health Assembly constitutes a strategic step forward “to strengthen vector control worldwide through increased capacity, improved surveillance, better coordination and integrated action across sectors and diseases” [3].

Following a call for proposals, in 2015, the current project was selected by WHO to produce six scoping reviews on the prevention and control of these diseases in urban areas [4]. Using a Delphi methodology that involved 109 international experts [5], the topics selected for these reviews were: 1) field validation and implementation of rapid diagnostic testing for vector-borne and other infectious diseases of poverty in urban areas [6]; 2) effective surveillance systems for VBDs in urban settings and translating the data into action [7]; 3) impact, economic evaluation, and sustainability of integrated vector management in urban settings to prevent VBDs [8]; 4) VBDs in urban areas: transmission dynamics, vectorial capacity, and co-infection [9]; 5) containment measures for emerging and re-emerging vector-borne and other infectious diseases of poverty in urban settings [10]; and 6) interventions for VBDs focused on housing and hygiene in urban areas [11]. The results of the six scoping reviews published in this special issue provide a clear picture of the available knowledge on each of the themes and highlight knowledge gaps, as well as needs and opportunities for further research. Based on the results of the scoping reviews, and to produce a list of priority research needs to be addressed in this area, the concept mapping method was used. This method has been used successfully in earlier experiences, for example, to develop conceptual frameworks [12], logic models [13], and measurement instruments [14], and to identify needs [15, 16]. concept mapping has also been used to identify research priorities [17], and authors of the present article (Dagenais, Ridde) have demonstrated its usefulness in this regard [18, 19].

With the recent international outbreak of Zika virus and associated cases of microcephaly [20], the media spotlight has been aimed on the seriousness of VBDs and the rapid spread of a little-known virus fuelled by rapid urbanization [21]. In this context of growing attention from research and international organizations on emerging and re-emerging VBDs, it is important to establish research priorities so that international collaborative efforts can be focused on the most urgent issues to rapidly improve the prevention and control of these diseases and their vectors. However, a literature search found no recent scientific publications (less than 3 years old) on collaborative process to determine research priorities. The purpose of the project highlighted in this report on in this special issue, therefore, is to provide rigorous evidence to inform research institutions and donor agencies about knowledge gaps and research priorities through an innovative three-step process (international eDelphi consultation, six scoping reviews, and concept mapping) that combines knowledge synthesis with the expertise and engagement of international public health researchers and decision-makers.

This article presents the final step in the process of identifying research priorities on VBDs and other diseases of poverty in the urban context. The previous steps are presented in various articles of this special issue [5–11]. To carry out this research prioritization exercise using the concept mapping method, members of the six research teams responsible for the “VEctor boRne DiseAses Scoping reviews” (VERDAS) consortium’s scoping reviews met for 2 days with decision-makers from Colombia, Brazil, and Peru, as well as with representatives from the Pan-American Health Organisation (PAHO) and WHO/the Special Programme for Research and Training in Tropical Diseases (TDR) .

## Methods

The concept mapping method developed by Trochim [22, 23] was adapted [24] and carried out using an analysis module specially designed by Provalis Research© (https://provalisresearch.com/). The technique organizes qualitative data using a series of statistical analyses. A total of 11 researchers and seven decision-makers completed the concept mapping exercise (Table 1). Data from three participants (two researchers and one decision-maker) had to be excluded because these individuals failed to classify more than 15 statements.

**Table 1:** Description of the participants

	Decision-makers^a^	Researchers	Total
Participants	8	13	21
Women	4	9	13
Men	4	4	8
Country of residence			
Brazil	3 (city, regional and federal levels)	4	7
Colombia	2 (regional and national levels)	4	6
Peru	1 (regional level)	0	1
Canada	0	3	3
France	0	1	1
Spain	0	1	1
USA (PAHO)	1	0	1
Switzerland (WHO/ TDR)	1	0	1
Training			
MD + PhD (public health or epidemiology)	3	5	8
PhD (all domains)	4	5	9
MD student	0	2	2
MSc	1	1	2
Specialty			
Surveillance, diagnostic and epidemiological	5	8	13
Entomology	2	1	3
Health economics	1	1	2
Community health	0	2	2
Virology	0	1	1

*MD* Doctor of medicine, *PhD* Doctor of philosophy, *Msc* Master of science

^a^For the purpose of describing participants, the representatives of PAHO and WHO/TDR are included in this category.

The concept mapping exercise was carried out in five steps over 2 days. Given the large number of scientific publications that have described this method (see, for example, the articles cited above), only the essential elements of the procedure are reported here.

1. First, participants generated a list of items during a brainstorming session to answer the question: “**In view of the knowledge synthesis (that you conducted) AND your own expertise, what do we still need to know about vector-borne diseases and other infectious diseases of poverty in urban areas?”** A list of 97 statements was produced during this first session.
2. The statements were then printed on individual cards and in list form. This material was given to each participant. Working individually, participants rated each of the 97 statements on two scales of 1 to 5 (5 being the most important); one scale was related to ‘priority’ and the other to ‘policy relevance’. Lastly, they sorted the statements, grouping cards together in piles based on their own individual criteria and expertise.
3. These data were entered into the software, after which statistical analyses (hierarchical cluster analysis [HCA], multidimensional scaling, average scores assigned to each of the items) were conducted to produce a preliminary map. HCA made it possible to produce any number of clusters, from 97, in which each statement would be its own ‘cluster’, to a single cluster grouping them all. The researchers in charge of the operation (Dagenais, Ridde, Degroote) then met to examine the content of the clusters produced and determine their optimal number. These decisions were based on a consensus from an empirical and heuristic standpoint. Besides a visual examination of the cluster contents and their relative importance, a statistical index provided information to help with interpretation. This was a specificity score, which is generally greater than 1, and which indicates the strength of an item’s association with a cluster. The higher the number, the more this item will be representative of the overall idea emanating from the cluster. Content analysis of the clusters continued until a final map emerged that everyone agreed was representative of the key dimensions. A 12-cluster solution was chosen, and conceptual labels were attached to each cluster based on the overall meaning of the constituent statements.
4. At another meeting these results were presented and discussed with all participants to ensure that the 12-cluster solution was agreed to by all participants.

## Results

**Fig. 1:**
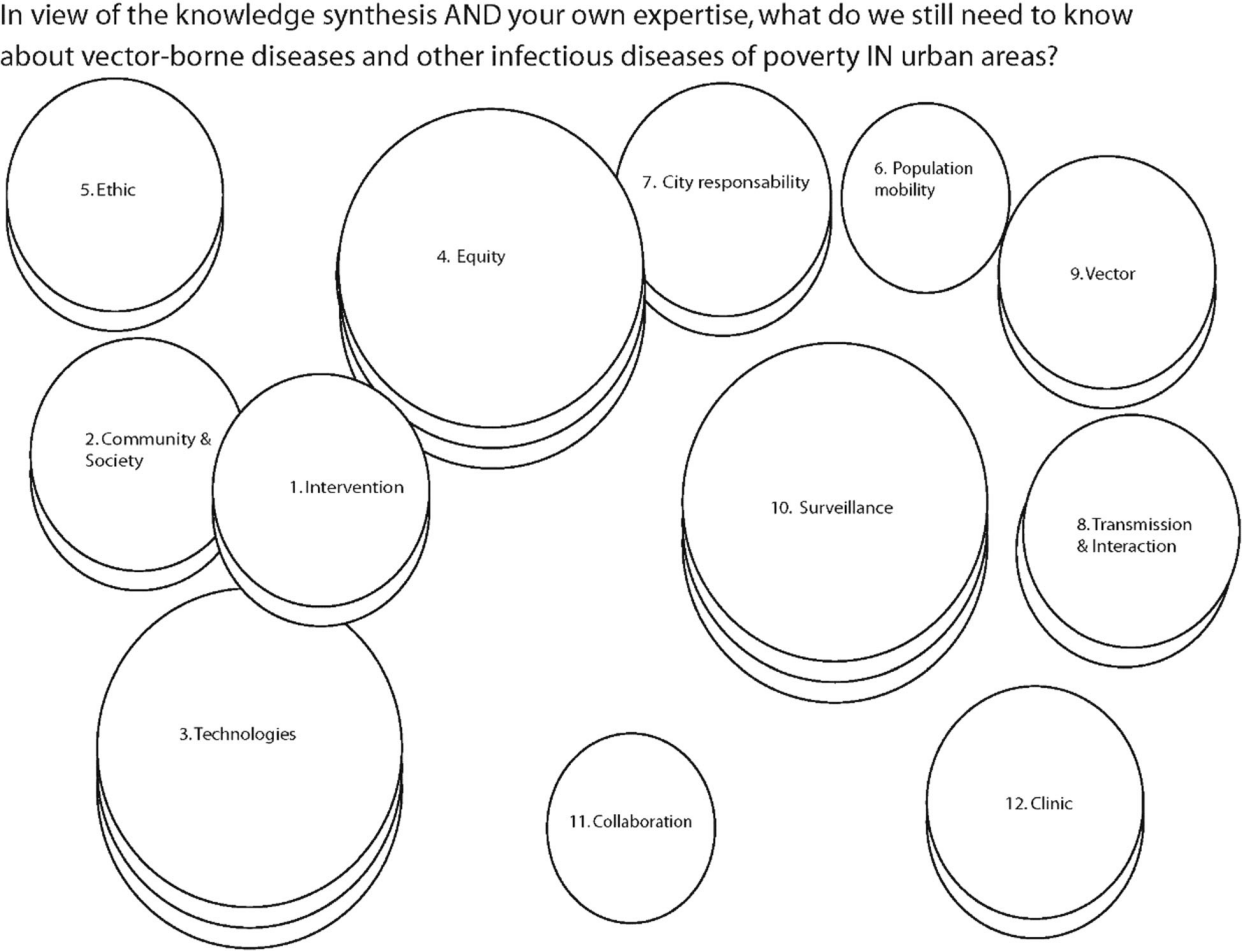
Research priorities determined by the collaborative concept mapping

The final map (Fig. 1) consists of 12 clusters whose size and number of strata represent the average importance of the statements making up the clusters. To determine the number of strata, the difference between the means of the highest and the lowest clusters was divided into three intervals. Thus, the “Technologies”, “Equity”, and “Surveillance” clusters were considered by participants to be the highest priority clusters. On the other hand, the “Population mobility” and “Collaboration” clusters were considered to be the lowest priority but remained identified by participants as research priorities. All other clusters were considered of medium importance.

The complete list of statements (ranked by importance ratings) for each cluster is presented in Additional file 2: Table S1. This table presents priority and policy relevance ratings for each item and averages for each cluster. These ratings are very similar for all clusters.

The average policy relevance scores of each of the 12 clusters is roughly the same as priority scores for all clusters. Only marginal differences are observed for the number of strata attributed to the “Intervention” and “Community & Society” clusters, which are considered more relevant and get one more stratum, while the “Vectors” and “Clinic” clusters get one less stratum on the policy relevance score.

The number of items per cluster is very heterogeneous, ranging from two items for the “Technologies” cluster to 23 items for the “Interventions” cluster. Based on priority scores, the most important cluster is the “Equity” cluster (3.83), which groups three items on integrating social determinants into the different facets of VBD research. The next cluster, “Technologies” (3.62), has only two items dealing with the integration of new technologies and their acceptability (including new vaccines). It should be noted that another item dealing with acceptability was placed in the “Interventions” cluster and concerns the consideration of social acceptability when designing interventions. In the “Surveillance” cluster (3.49), which consists of 16 items, a strong need was identified for knowledge around the detection of epidemics. Items include the need to improve surveillance systems in the light of recent developments (new technologies, big data, and geographical information) and scaling up and harmonizing these systems and protocols at the national and international levels to foster better international collaboration. Some items also focus on the performance of systems to identify the most vulnerable areas and on the use and transfer of information to decision-makers. With respect to the 23 items in the “Interventions” cluster (3.45), most knowledge needs identified by participants relate to evaluating interventions, including the integration of multilevel analyses (individual, community, population) and the improvement of effectiveness measures for different types of interventions. Several items deal with research on the implementation of interventions and, in particular, on the integration of interdisciplinary and multi-disease approaches and sustainability. This result highlights the complexity of the interventions. Finally, the issue of the roles of industry and governments in the procurement of vector control tools is highlighted. The “Ethics” cluster (3.44) contains three rather vague items on the need to identify the different ethical dimensions associated with research on VBDs. In the “Transmission & Interaction” cluster (3.37), the nine items summarize knowledge needs for a better understanding of different transmission routes, of co-circulation and co-infection of different viruses, and of the factors associated with lethality and congenital syndromes. The “Community & Society” cluster (3.31) grouped seven items on the role and capacity-building of communities to address VBDs, as well as on education and training issues for children and health professionals. The “Vectors” cluster (3.26) contains seven items covering, on one hand, the biology and behaviour of vectors and, on the other, the use of entomological indices for predicting epidemics and their limitations. The “City responsibility” cluster (3.20) includes nine items on the impact of urban development and how to integrate the fight against VBDs into sustainable urban planning (including waste management and sanitation issues). This cluster also includes questions about collaboration between the various municipal departments and the impact of climate change. In the “Clinics” cluster (3.16), the vast majority of the 10 items focus on the development, use, and validation of rapid diagnostic tests (RDTs) and other diagnostic methods, including the need for biomarkers. The four items in the “Collaboration” cluster (3.08) focus on how to make research more responsive to emergencies and health priorities. Finally, the “Population mobility” cluster (2.71), with four items, deals with evaluating the role of mobility in the spread of VBDs and the preventive means that could be used. Participants gave this issue the lowest priority.

Table 2 provides a list of the statements with the highest ratings on both priority and policy relevance. These 10 statements fall into six different clusters: “Interventions”, “Community & Society”, “Equity”, “Ethics”, “City responsibility”, and “Surveillance”. It should be noted that four of them come from the “Interventions” cluster. The average importance of the statements in this cluster was re-calculated without including these four statements. The results showed that they did not influence the number of strata allocated to this cluster. In other words, they did not inflate the mean value of this category. This is not surprising given the large number of items in this cluster. Table 2 also shows that the average scores attributed to priority and policy relevance are generally very close together and none exceeds half a point. In other words, for the concept mapping participants, research priorities were also policy-relevant.

**Table 2:** Statements with the highest “Priority” and “Policy relevance” ratings

No.	Statements	Cluster	Priority	Policy relevance
3	The effectiveness of integrated vector control management	Interventions	4.28	4.56
1	What determines the success, effectiveness, and sustainability of preventive strategies	Interventions	4.06	4.22
26	What surveillance systems are needed to predict the next outbreaks of VBDs	Surveillance	4.06	4.00
18	How to apply the social determinant approach in integrated vector management	Equity	3.94	3.94
22	What are the impacts of interventions on health outcomes at the community level	Interventions	3.94	3.94
57	What are the ethical dimensions we need to take into account in interventions	Ethics	3.89	4.00
84	How to take into account equity in surveillance and in interventions	Equity	3.89	4.00
13	What are the sanitation waste management strategies that can help prevent VBDs	City responsibility	3.83	4.22
54	How to take social acceptability into account when designing an intervention	Community & Society	3.83	4.28
79	Barriers and facilitators for environmental sustainability of integrated vector management	Interventions	3.78	4.17

*VBDs* Vector-borne diseases

Finally, Table 3 presents the 10 statements with the lowest priority and policy relevance ratings. They are divided into seven different clusters: “Ethics”, “Population mobility”, “City responsibility”, “Transmission & Interaction”, “Vectors”, “Collaboration”, and “Clinics”. Two come from the “Vectors” cluster and three from the “Clinics” cluster. However, even if these items had been excluded, the average scores for these two clusters would not have reduced their relative importance significantly enough to move to a single stratum in the final map (Fig. 1). As with the top 10 items, there is no significant difference between the priority and policy relevance ratings.

**Table 3:** Statements with the lowest priority and policy relevance ratings

No.	Statements	Cluster	Priority	Policy relevance
78	How important is the case management of VBDs in transmission?	Transmission & Interaction	2.89	2.72
52	Is it possible/ethical to treat people in order to kill the mosquitoes?	Ethics	2.83	2.33
49	How many RDTs do we need for a single disease?	Clinics	2.67	2.28
86	What is the role of multiple testing at the point of care?	Clinics	2.67	2.89
73	What is the current state of RDTs for leptospirosis?	Clinics	2.56	2.50
34	Can low vector infestation (house index) cause outbreaks in large urban settings?	Vectors	2.50	2.56
82	What are the best patterns to evaluate vector capacity and unexpected species?	Vectors	2.50	2.44
77	Are resources, networking possible in VBD research?	Collaboration	2.50	2.11
8	What do we mean by urban areas?	City responsibility	2.33	2.11
46	How can we prevent people with viremia from moving around?	Population mobility	1.50	1.94

*RDTs* Rapid diagnostic tests, *VBDs* Vector-borne diseases

## Discussion

The starting point for this concept mapping exercise was the work of six research teams that carried out six scoping reviews on selected themes following an extensive expert consensus technique using the Delphi method. Based on this work, and from a participatory standpoint, the aim of the concept mapping exercise was to identify, in a collaborative process involving researchers from these teams and high-level decision-makers, the research themes for which studies should be carried out as a priority.

Identifying research priorities is a major challenge for researchers and potential users of research results. Prioritization approaches use a variety of more or less structured methods: a working group to take stock of the state of knowledge and recommend research activities to be prioritized [25]; broad unstructured consultations of international experts [26]; meeting of specialists to gather their views and experiences on the topic under consideration [27, 28]; and conducting extensive polling surveys [29], combined with focus groups [30]. Some activities of the same type use more structured methods, such as the one developed by the Child Health and Nutrition Research Initiative Method, in which several experts generate and score research options against a set of criteria [31–33]; others use the nominal group technique [34]. To determine the topics of the six scoping reviews in this project, the Delphi technique was used [35, 36].

### Priority research areas

The cluster considered the most important concerns equity issues, confirming that these issues are rarely addressed in research on VBDs. As is often the case in public health, equity is set aside for research on the effectiveness of interventions [37]. However, when it comes to VBDs, the poorest communities and individuals are often the most affected [38], with an even more unbalanced distribution of risk among children, women of childbearing age, and the elderly [38–41]. Yet, programs and policies for VBD prevention and control are still all too often designed to meet the needs of the population as a whole without taking into account the unequal distribution of risks among this population [42]. The fight against Zika was not seized by health actors as an opportunity to fight against injustices and promote equity [43]. As the 10th anniversary of the WHO Commission report [44] on the social determinants of health is celebrated, this study shows that the needs for equity are still enormous. For example, a few interventions to combat VBDs have been developed with a view to achieving proportionate universalism, as widely recommended today [45]. This concept mapping exercise therefore shows that, in the fight against VBDs, equity issues must now be front-and-centre, and that more research projects should be developed to understand how to integrate social determinants more effectively into public health programs and policies.

The second and third highest priority clusters are about new technologies and surveillance. These are fundamental issues. Once again, recent international epidemics have highlighted the strengths and limitations of current surveillance systems [46] and the need for early detection of outbreaks [47]. An effective surveillance system should collect and analyze reliable data to produce relevant information for sharing with those who can promote new public health policies and implement prevention and control strategies [48]. Today, however, several of these elements are still lacking or need to be improved, particularly by using new technologies, as was attempted in Burkina Faso by members of our team [49]. The Ebola crisis in West Africa has shown that surveillance systems are not always adequate [50]. As mentioned in the scoping review on surveillance systems, there is an important need for innovative research to take into account the ever-expanding environmental, social, and health changes in urban areas [7]. From the concept mapping exercise and conversations among participants, it also emerged that one of the top research priorities is to identify relevant and realistic thresholds for early detection of epidemics and to support the implementation of control actions. As such, in a context of rapid technological development, it is important to fund initiatives that integrate new information and communications technologies (ICTs) to improve surveillance systems, such as the use of smartphone applications and services [51, 52] or big data (geographic and demographic) [53, 54].

Although no bioethicist was included in the group of participants, which is regrettable, the issue of ethics in the fight against VBDs emerged as a research priority. The aim is to gain a better understanding of the ethical considerations to be taken into account, particularly in prevention and control interventions. As mentioned, the ethical issue was approached from the perspective of social justice and equity. It is therefore not only a question of traditional procedural ethics, but also of how interventions can be formulated and implemented in this ethical perspective [55, 56]. A list of criteria public health actors could apply when considering the ethical aspects of their interventions was also proposed [57].

Intervention research is another priority, particularly on vector control interventions. As shown in the scoping reviews on containment measures [10] and household prevention [11], it is somewhat surprising to note how little is known about the effectiveness and implementation of interventions when the critical role vectors play in VBD transmission has long been known. There is a critical need for funding and publications of complex interventions of high methodological quality in order to provide the evidence necessary for decision-makers to implement new public health policies [58]. There is also an urgent need to improve the methods of evaluating interventions, which are all too often simplistic, whereas interventions are increasingly complex and multidisciplinary [11]. Innovative approaches based on validated theories and mixed methods are urgently needed [59–61]. The problem of the social acceptability of interventions has been highlighted, and this question has still not been sufficiently studied by researchers. Recent methodological advances [62, 63] will be useful for empirical testing in this field. Moreover, the research on interventions that comply with methodological gold standards is very costly and requires significant funding over several years. It is therefore important to have a real investment from the scientific and political communities and funding agencies to provide solid evidence quickly to guide future public health policies [58].

### Issues not mentioned during the concept mapping

The list of items proposed during the brainstorming was, of course, influenced by the expertise of the participants present and the preparatory work of the six scoping reviews, whose topics were chosen by the eDelphi panel from more than 120 proposals. It is obvious that the list of items could not be exhaustive, and the subjects of the six scoping reviews are very well represented (interventions, surveillance, diagnosis, transmission). It is therefore also interesting to revisit some topics that were not mentioned during the concept mapping but were raised in the eDelphi consultation at the beginning of the project. Governance issues were not addressed during the concept mapping, nor were access and quality of care. Issues related to the development and distribution of new vaccines were mentioned in a single item on the acceptability of these new treatments. There was also very little discussion of antibiotic and pesticide resistance during the exercise, and so this was not directly reflected in the research priorities identified, although it underlies several themes discussed (particularly in the clusters on interventions and new technologies). The issue of costs and economic analysis did not appear as a separate theme but was integrated into the cluster on interventions. Economic issues were therefore not identified as a research priority as such, but should be given greater consideration in the design and analysis of future interventions. Public policies, sustainability, the institutionalization of vector control, and the role of intersectorality were poorly noted during the Delphi consultation and did not appear as research priorities during the concept mapping exercise. Finally, it was surprising that the issue of climate change appeared only twice in the concept mapping and was not more preponderant, given that it is at the heart of contemporary debates. [48]. All these issues would certainly have been raised if the panel had included economists, climatologists, vaccinologists, or even experts in health systems.

### Strengths and limitations

The concept mapping method used has many strengths and benefits. Previous work from authors with concept mapping [17, 18, 23, 63–65] and their review [66] of 190 articles published since 1989 show that it: 1) combines qualitative and quantitative data; 2) provides images or configurations that simultaneously represent the main concepts, ideas, phenomena or dimensions at stake and their relative importance; 3) requires relatively few resources and is achieved in a few days only; 4) has many benefits for participants (sense of cohesion, belonging, discussion and sharing of opinions and ideas); 5) produces useful results, in the language of the participants and that 6) the participants appreciate the collaborative process; 7) provides equal weight to the views of each participant in the statistical calculations; and 8) helps to minimize the researchers’ biases. However, this method also presents its share of difficulties and limitations, such as: 1) poor external validity, since it is not clear whether the results produced apply to other areas or problems; 2) difficulty in forming representative groups; 3) constraints imposed during brainstorming (formulation of statements related to a specific issue, without the opportunity to discuss or debate), which can cause frustration; 4) the need to recruit an experienced facilitator; 5) the difficulty of formulating a clear and unambiguous question that covers the entire field explored.

This exercise brought together participants from a variety of disciplines, countries, and organizations. Regrettably, there were no decision-makers from Africa or Asia, although researchers working in these regions were present. The approach made the identified research priorities more relevant, but at the same time may have introduced some bias in favour of intervention and surveillance research, as the researchers and decision-makers participating were particularly active in this area. These are the clusters with the greatest number of items, and while this certainly corresponds to priority research needs, it is also possible that this may reflect the networks of researchers who took the initiative to respond to the WHO call [59].

## Conclusions

The purpose of this concept mapping exercise was to determine research priorities in the control and prevention of VBDs in urban areas. This collective exercise identified 12 categories of themes for which research still provides too few answers. The question now is how research funding agencies and researchers in the field will use the results of this exercise to guide future work. The challenge is to put forward a knowledge transfer strategy for this project. With this in mind, the study protocol submitted to the WHO call included a plan to produce a policy brief for each scoping review, addressed to potential users of these results. To prepare these briefs, a member of the team, a knowledge transfer specialist, provided training and coaching. These briefs, written in plain language and presented in an attractive format, will summarize the results of the scoping reviews and propose operational recommendations for their implementation. In the view of the authors, these transfer tools may be useful, but only to the extent that they are included in a structured knowledge transfer mechanism.

## Additional file

Additional file 1: Multilingual abstracts in the six official working languages of the United Nations. (PDF 491 kb)

Additional file 2: **Table S1.** List of statements in each cluster sorted by priority and policy relevance ratings. (DOCX 24 kb)

## Abbreviations

HCA: Hierarchical cluster analysis
ICTs: Information and communications technologies
PAHO: Pan-American Health Organisation
TDR: The Special Programme for Research and Training in Tropical Diseases
VBDs: Vector-borne diseases
WHO: World Health Organization
