# Supplementary material for: Establishing research priorities in prevention and control of vector-borne diseases in urban areas: a collaborative process
Source: Infect Dis Poverty. 2018 Sep 3;7:85. doi: 10.1186/s40249-018-0463-y (PMC6120077; doi:10.1186/s40249-018-0463-y)
Supplement: Supplementary file 1 — Multilingual abstracts in the six official working languages of the United Nations. (PDF 491 kb) [file 40249_2018_463_MOESM1_ESM.pdf]

Translation of the abstract into the six official working languages of the United Nations

ةينواعت ةيلمع :ةيرضحلا قطانملا يف اهتحفاكمو لقاونلاب ةلوقنملا ضارمألا نم ةياقولا لاجم يف ثحبلا تايولوأ ءاسرإ

ديرِ يريلاف ،اويامات-زيدوميرب ارالك ،وييرابال يد ينامثع ميرم ،تورغيد ينافيتس ،سيانيغاد نايتسيرك

صخلم

ةيفلخلا: في عام 2015، عقب دعوة لتقديم مقترحات من برنامج الخاص للبحوث والتدريب في مجال أمراض المناطق المدارية، (تي دي آر) 6 مراجعات استطلاعية حول الوقاية من الأمراض المنقولة بالنواقل ومكافحتها في المناطق الحضرية. توفر تلك المراجعات صورة واضحة عن الحالة الراهنة للمعرفة، وتسلط الضوء على الفجوات في المعرفة والاحتياجات والفرص للبحوث المستقبلية. واستناداً إلى النتائج الجديدة للمراجعات الاستطلاعية، تم اتخاذ خارطة مفاهيم من أجل إعداد قائمة بالبحوث ذات الأولوية التي ينبغي معالجتها.

الطريقة: جماعة علماء من فرق البحث المسؤولين عن المراجعات الاستطلاعية الست حول الأمراض المنقولة بالنواقل (VEctor-borNe DiseAses Scoping (VERDAS) لمدة يومين مع صانعي القرار من كولومبيا والبرازيل وبيرو ومنظمة الصحة للبلدان الأمريكية ومنظمة الصحة العالمية. أكمل ما مجموعه 11 باحثاً و سبعة من صانعي القرار (من وزارات الصحة، ومديرية مكافحة النواقل والأمراض في المدن والمناطق، ومبرمج مكافحة النواقل والأمراض) رسم خرائط المفاهيم حيث أجابوا على السؤال التالي: "نظراً لخبرتك/معرفتك الشخصية، ما هي الأمور التي لا تزال بحاجة إلى معرفة أن بشأن الأمراض المنقولة بالنواقل وأمراض الفقر الأخرى في المناطق الحضرية؟" قام المشاركون بترتيب البيانات في مجموعتين استناداً إلى المعايير "الأولويات" و"ملاءمة السياسات"، وجمع في جميع البيانات على مقياس من 1 إلى 5، أحدهما يتعلق بالخبرة والمعرفة.

النتائج: تألفت الخريطة النهائية من 12 مجموعة. اعتبر المشاركون أن تلك التي تتعلق بـ"الإنصاف" و"التكنولوجيا" و"المراقبة" أولويات قصوى. إن الإنصاف هي الأهم وذلك يؤكد أننا نادراً ما يتم التعامل مع هذه القضايا في البحوث المتعلقة بالأمراض المنقولة بالنواقل. على الرغم من أن مجموعتي "التنقل" و"التعاون" هي الأدنى من حيث الأولويات، إلا أن المشاركين حددوها كأولويات بحثية. وكانت متوسط درجات الملاءمة للسياسات في كل مجموعة من المجموعات الاثنتي عشرة هو نفس درجات الأولويات. لم يتم تناول بعض القضايا - مثل الحوكمة والوصول إلى الرعاية ونوعيتها - خلال عصف ذهني.

الاستنتاجات: استناداً إلى هذا العمل، والاعتماد على نهج تشاركي، تم إجراء عملية رسم خرائط المفاهيم بالتعاون مع الباحثين من هذه الفرق. حدد صانعو القرار الدراسات والأبحاث التي ينبغي إجراؤها في المستقبل.

Translated from English version into Arabic by Ammar Alhosain, proofread by Hiba Trefi, through

确定城市地区媒传疾病的防治重点：一项协作研究

Christian Dagenais, Stéphanie Degroote, Mariam Otmani Del Barrio, Clara Bermudez-Tamayo, Valéry Ridde

摘要

引言：2015 年在热带病培训研究特别规划署（TDR）征集提案后，确定撰写 6 篇涉及城市地区媒传疾病防治的勘域综述。这些综述清楚地介绍了当前进展，突出了知识差距，以及未来研究的需求和方向。根据上 述勘域综述的研究结果，完成概念图，编制了一份优先研究的需求清单。

方法：负责媒传疾病勘域综述的 6 个研究小组成员与来自哥伦比亚、巴西、秘鲁、泛美卫生组织和世界卫生组织的决策者进行了为期 2 天的会面。共有 11 名研究人员和 7 名决策者（来自卫生部门、城市和区域病媒控制部门以及病媒控制项目）参与完成概念图，并回答了以下问题："鉴于您的专业和综合知识，我们 是否还需要了解城市地区的媒传疾病和其他贫困所致传染病？"。参与者将每份报告分为 2 类，评分均为 1 到 5，一类为"优先级别"，另一类是"政策相关性"，并根据各自的标准和专业知识提交评分结果。

结果：最后的概念图由 12 个集群组成。参与者认为"公平"、"技术"、"监测"集群的优先级最高。在所有集群中，最重要的是公平问题，但在媒传疾病研究中很少涉及这些问题。另一方面，尽管"人口流动"和"协作"集群的优先级最低，但仍被参与者确定为研究重点。12 个集群中，每个集群的平均策略相关性得分与其优先级得分大致相同。有些问题在头脑风暴中未能解决，如治理、获取和护理质量就属于这种情 况。

结论：本研究通过参与式方法，与团队研究人员和高级决策者合作完成概念图，确定了应优先开展的研究课题。

Translated from English version into Chinese by Fan Yang, edited by Pin Yang

Établir les priorités de recherches dans la prévention et le contrôle des maladies à transmission vectorielle dans les zones urbaines: processus de collaboration

Christian Dagenais, Stéphanie Degroote, Mariam Otmani Del Barrio, Clara Bermudez-Tamayo et Valéry Ridde

Résumé

Contexte: En 2015, à la suite d'un appel à proposition du Programme spécial de recherche et de formation concernant les maladies tropicales (TDR), six examens de la portée sur la prévention et le contrôle des maladies à transmission vectorielle en milieu urbain ont été réalisés. Ils ont mis en évidence les connaissances disponibles et les lacunes à combler, mais aussi les besoins et les possibilités de recherches à venir. Basé sur les résultats de ces examens, une opération de cartographie conceptuelle a été entrepris afin d'établir une liste des besoins de recherche prioritaires à traiter.

Procédés: Les membres des six équipes de recherche en charge des examens de la portée menés par le groupe VERDAS se sont concertés durant deux jours avec des décisionnaires venant de la Colombie, du Brésil, du Pérou, de l'OPS et de l'OMS. Au total, 11 chercheurs et sept décisionnaires (provenant de ministères de la santé, des services de contrôle des vecteurs municipaux et régionaux, et des programmes de lutte antivectorielle) ont participé à un remue-méninges pour répondre à la question: «À la lumière de cette synthèse de connaissances et de notre propre expertise, que nous reste-t-il à découvrir concernant les maladies à transmission vectorielle et autres maladies infectieuses dues à la pauvreté en milieu urbain?» Les intervenants ont évalué chaque énoncé sur deux échelles allant de 1 à 5 : l'une concernant la priorité, l'autre l'intérêt pratique, puis ils ont regroupé ces énoncés en différentes catégories en fonction de leurs différents critères et expertises.

Résultats: La carte finale est composée de 12 catégories. Les intervenants ont considéré les catégories "Équité", "Technologie" et "Contrôle" comme étant les plus prioritaires. La catégorie jugée la plus importante est celle traitant des problèmes d'équité, ce qui confirme que ces problèmes ne sont que rarement traités dans le cadre de la recherche sur les maladies vectorielles. En outre, les catégories "Mobilité de la population" et "Collaboration" ont été considérées comme étant les moins prioritaires mais restent, néanmoins, listées comme priorités en matière de recherche. La cote d'importance moyenne de l'intérêt pratique des 12 catégories reste approximativement la même que la cote de priorité pour chacune d'entre elles. Certains problèmes n'ont pas été abordés durant cette concertation. Cela a été le cas du problème de gestion et celui de l'accès aux soins et de leur qualité.

Conclusions: Se fondant sur ces réflexions et adoptant une approche participative, le processus de cartographie conceptuelle entrepris conjointement par ces chercheurs et ces décisionnaires de haut niveau a permis de définir les thèmes de recherche qui devront être abordés en priorité.

Translated from English version into French by William Squire, proofread by Ode Laforge, through

Определение приоритетов для исследований по профилактике и контролю за распространением заболеваний, передаваемых переносчиками, в городских районах: процесс сотрудничества

Кристиан Дежене, Стэфани Дегрут, Мариам Отмани Дель Баррио, Клара Бермудес-Тамайо, Валери Ридд

Аннотация

Общие сведения: В 2015 году по запросу Специальной программы по научным исследованиям и подготовке специалистов в области тропических болезней (СПТБ) было проведено шесть предварительных исследований по профилактике и контролю за распространением заболеваний, передаваемых переносчиками, в городских районах. Эти исследования дали четкое представление о доступной

информации и выявили пробелы в знаниях, а также потребности и возможности будущих исследований. На основе результатов предварительных исследований был составлен проект концепции и список приоритетных направлений исследований.
Методы: Ученые из шести групп, ответственных за подготовку обзорных статей в рамках консорциума VERDAS, провели двухдневную встречу с представителями Колумбии, Бразилии, Перу, ПАОЗ и ВОЗ. 11 ученых и семь представителей министерств здравоохранения, городских и региональных департаментов по борьбе с переносчиками заболеваний и программ по борьбе с переносчиками заболеваний составили проект концепции, отвечая на вопрос: «С учетом накопленного объема знаний и вашего личного опыта, что еще необходимо узнать о заболеваниях, передаваемых переносчиками, и других инфекционных заболеваниях, сопутствующих бедности в городских районах?» Участники оценили каждое утверждение по двум шкалам от 1 до 5 (по шкале приоритетности и по шкале политической актуальности) и сгруппировали утверждения в кластеры, исходя из собственных индивидуальных критериев и опыта.
Результаты: Полученный в итоге проект состояла из 12 разделов. К числу наиболее приоритетных участники отнесли такие разделы как «Справедливость», «Технологии» и «Наблюдение». Самым важным сочли раздел, посвященный вопросам справедливости, поскольку этим вопросам редко уделяется внимание в рамках исследований заболеваний, передаваемых переносчиками. В свою очередь, разделы «Мобильность населения» и «Сотрудничество», хоть и были признаны наименее приоритетными, были оставлены участниками в числе приоритетов для исследований. Средняя оценка каждого из 12 разделов по шкале политической актуальности примерно совпадала с оценкой всех разделов по шкале приоритетности. Некоторые вопросы в ходе мозгового штурма не рассматривались. Например, вопросы управления, а также доступа к медицинской помощи и ее качества.
Выводы: Составленный по итогам совместной работы в рамках сотрудничества ученых и высокопоставленных чиновников проект концепции позволил выявить приоритетные темы для дальнейших исследований.

Translated from English version into Russian by Daria, proofread by Oksana Weiss, through

TRANSLATORS
WITHOUT BORDERS

Establecimiento de las prioridades de investigación en materia de prevención y control de las enfermedades transmitidas por vectores en zonas urbanas: un proceso colaborativo.

Christian Dagenais, Stéphanie Degroote, Mariam Otmani Del Barrio, Clara Bermudez-Tamayo, Valéry Ridde

Resumen
Antecedentes: En 2015, tras una convocatoria de propuestas del Programa Especial de Investigación y Capacitación sobre las Enfermedades Tropicales (TDR, sus siglas en inglés), se presentaron seis estudios de alcance sobre la prevención y el control de las enfermedades transmitidas por vectores en zonas urbanas. Estos estudios proporcionaron una visión nítida del conocimiento disponible; sin embargo también destacaron la falta de conocimientos, así como las necesidades y las oportunidades para investigaciones futuras. Basándose en los hallazgos de las investigaciones de los estudios de alcance, se llevó a cabo un mapa conceptual con el fin de elaborar una lista de necesidades prioritarias de investigación que deben abordarse.
Métodos: Durante dos días, miembros de los seis equipos de investigación responsables de los estudios de alcance del Consorcio VERDAS se reunieron con autoridades provenientes de Colombia, Brasil, Perú, la Organización Panamericana de la Salud (OPS) y la Organización Mundial de la Salud (OMS). Un total de once investigadores y siete autoridades (procedentes de ministerios de la salud y de departamentos de control de vectores municipales y regionales) completaron el mapa conceptual y respondieron a la siguiente pregunta: "Dada esta síntesis de conocimientos y basándose en su propia experiencia, ¿qué nos falta aún por saber sobre las enfermedades transmitidas por vectores y otras enfermedades infecciosas relacionadas con la pobreza en zonas urbanas?". Los participantes valoraron del 1 al 5 cada afirmación de acuerdo a dos escalas, una relativa a la "prioridad" y otra relacionada con su "relevancia política". A continuación, reagruparon las afirmaciones basándose en sus propios criterios y experiencias personales.

Resultados: El mapa final estaba compuesto por 12 grupos. Los participantes consideraron que los grupos denominados "Equidad", "Tecnología" y "Supervisión" poseían la máxima prioridad. El grupo considerado como el más importante está relacionado con temas de equidad, lo que confirma que estas cuestiones rara vez se abordan en las investigaciones sobre las enfermedades transmitidas por vectores. Por otro lado, se consideró que los grupos denominados "Movilidad de la población" y "Colaboración" tenían la prioridad más baja, pero los participantes continuaron identificándolos como prioridades de investigación. Las puntuaciones medias referentes a la importancia política para cada uno de los 12 grupos fueron prácticamente las mismas que las calificaciones de prioridad para todos los grupos. Algunas cuestiones no se abordaron durante el intercambio de ideas. Es el caso del tema de la gobernabilidad y el acceso y la calidad de la atención.

Conclusiones: Basándose en este trabajo y adoptando un enfoque participativo, el mapa conceptual llevado a cabo de forma colaborativa por investigadores de estos equipos y algunas autoridades de alto nivel han identificado temas de investigación prioritarios que deben ser objeto de estudios futuros.

Translated from English version into Spanish by Carolina, proofread by Maribel, through

TRANSLATORS
WITHOUT BORDERS
