# Supplementary material for: Establishing research priorities in prevention and control of vector-borne diseases in urban areas: a collaborative process
Source: Infect Dis Poverty. 2018 Sep 3;7:85. doi: 10.1186/s40249-018-0463-y (PMC6120077; doi:10.1186/s40249-018-0463-y)
Supplement: Supplementary file 2 — Table S1. List of statements in each cluster sorted by priority and policy relevance ratings. (DOCX 24 kb) [file 40249_2018_463_MOESM2_ESM.docx]

Additional file 2: Table S1 List of statements in each cluster sorted by priority and policy relevance ratings

| **No.** | **Statements** | **Priority** | **Policy relevance** |
| --- | --- | --- | --- |
| Cluster | **Equity** | **3,83** | **3,89** |
| **18** | How to apply the Social Determinant approach in Integrated Vector Management | 3,94 | 3,94 |
| **84** | How to take into account equity in surveillance and in interventions | 3,89 | 4 |
| **35** | What are the diffenrential burden in VBDs attributable to health inequality/social determinants | 3,67 | 3,72 |
|  | | | |
| Cluster | **Technologies** | **3,62** | **3,72** |
| **95** | Acceptability/adoption of vaccines / (new) drugs and other health technologies to prevent VBDs | 3,67 | 3,72 |
| **44** | How to use new technologies to control VBDs | 3,56 | 3,72 |
|  | | | |
| Cluster | **Surveillance** | **3,49** | **3,62** |
| **26** | What surveillance systems are needed to predict the next outbreaks of VBDs | 4,06 | 4 |
| **71** | How can we use geographic information systems to improve surveillance | 3,72 | 3,83 |
| **14** | Do we need a structured risk assessment approach/tool to identify vulnerable areas | 3,67 | 3,94 |
| **15** | If we need a structured risk assessment approach/tool to identify vulnerable areas, what should be the components of this tool? | 3,67 | 3,67 |
| **66** | What are the barriers and facilitators to use surveillance data by decision-makers | 3,67 | 4,28 |
| **32** | How can surveillance system/interventions be scaling up and replicated across countries | 3,67 | 4,11 |
| **25** | How could we use better transfer the information from surveillance systems | 3,61 | 4,06 |
| **28** | How do we use big data to improve the control of arbovirus infection | 3,56 | 3 |
| **81** | Is it necessary to harmonize protocols to generate epidemiological parameters for VBDs | 3,50 | 3,5 |
| **69** | Which are the appropriated indicators to evaluate risk | 3,44 | 3,22 |
| **96** | How to harmonize modelling methods to be more relevant to reality | 3,44 | 2,78 |
| **88** | Which are the outbreaks predictors and detection at country response | 3,33 | 3,89 |
| **43** | How to improve the design of routine entomological surveys in order to predict epidemics | 3,33 | 3,61 |
| **38** | How he outbreaks or epidemic of arbovirus impact the surveillance of other diseases | 3,22 | 3,39 |
| **74** | How can we improve the experience sharing between countries in term of interventions | 3,22 | 3,56 |
| **59** | Other components to disease and entomological surveillance are required for disease control | 2,72 | 3,11 |
|  | | | |
| **Cluster** | **Interventions** | **3,45** | **3,51** |
| **1** | What determine the success, effectiveness and sustainability of preventive strategies | 4,06 | 4,22 |
| **3** | The effectiveness of integrated vector control management | 4,28 | 4,56 |
| **90** | What is the effectiveness of use of bednets programs for Aedes transmission diseases | 3 | 3,06 |
| **6** | What are the most appropriated approaches that should be use in IVM control in order to organize | 2,94 | 2,94 |
| **47** | How to include a multi-diseases approach for integrated vector management within one strategy of control | 3,39 | 3,61 |
| **94** | Comparative studies about externalities and consequences about the main vector control strategies and tools | 3,17 | 3,28 |
| **29** | Assess new integrated vector control strategies able to limit insecticide resistance | 3,72 | 3,78 |
| **79** | Barriers and facilitators for environmental sustainability of integrated vector management | 3,78 | 4,17 |
| **2** | What are the outcomes that I should expect + or – from implemented strategies to control VBDs | 3,61 | 3,78 |
| **22** | What are the Impacts of interventions on health outcomes at the community level | 3,94 | 3,94 |
| **10** | Methods needed to target interventions | 3,72 | 3,39 |
| **62** | How can we take into account the complexity of the interventions | 2,89 | 2,78 |
| **23** | How to improve the designs of the interventions based on theories | 3,33 | 2,61 |
| **97** | We need to emphasize on high quality methodological standards while reporting interventions | 3,78 | 3,17 |
| **55** | Is economic approach an adequate tool to assess effectiveness of VBDs interventions? | 2,72 | 3,06 |
| **56** | What are the components we need to have to | 3,39 | 3,44 |
|  | decide the effectiveness of an intervention? |  |  |
| **92** | How can interventions be better designed to establish causal effectiveness | 3,78 | 3,5 |
| **45** | How to implement interventions based on proportional universalism | 2,94 | 3,17 |
| **33** | How to improve interdisciplinary research on implementation interventions | 3,78 | 3,28 |
| **60** | What simple implementation research methods can be included in routine public health | 3,5 | 3,83 |
| **11** | What is the role of the industry in providing vector control tools including sanitation and waste management | 2,83 | 2,89 |
| **16** | What of the current global public health policies effective, and why? | 3,61 | 4,06 |
| **42** | What are the roles of state in provision of tools for prevention of VBDs | 3,17 | 4,11 |
|  | | | |
| Cluster | **Ethics** | **3,44** | **3,24** |
| **57** | What are the ethical dimensions we need to take into account in interventions | 3,89 | 4 |
| **68** | What are the ethical considerations in vector control strategies | 3,61 | 3,39 |
| **52** | Is it possible/ethical to treat people in order to kill the mosquitoes | 2,83 | 2,33 |
|  | | | |
| Cluster | **Transmission & Interaction** | **3,37** | **3,06** |
| **19** | Are there any other factors beside Zika infection associated with congenital syndrome | 3,78 | 3,72 |
| **64** | Which factors are associated with death caused by arbovirus infections in different context | 3,78 | 3,67 |
| **51** | The role of different transmission routes for the maintenance of Zika infection spread | 3,50 | 3,11 |
| **27** | What are the roles of the interaction/coinfections between infectious agents on the modelling of the transmission | 3,39 | 2,5 |
| **63** | What co circulation impact the pattern of severity and other complications of arbovirus | 3,39 | 3,11 |
| **53** | The role of different transmission routes on the clinical outcomes of Zika | 3,28 | 2,83 |
| **91** | To determine the influence of new flavivirus emergence in dengue vaccine evaluation | 3,28 | 2,89 |
| **87** | Which conditions How to avoid the introduction of new agents arbovirus in open area where arbovirus are circulated | 3,06 | 3 |
| **78** | How important is the case management of VBDs in transmission | 2,89 | 2,72 |
|  | | | |
| **Cluster** | **Community & Society** | **3,31** | **3,63** |
| **54** | How to take social acceptability into account when designing an intervention | 3,83 | 4,28 |
| **30** | How to facilitate community capacity building in control of VBDs | 3,61 | 3,78 |
| **39** | Can school education can provide effective to tools to prevent/interrupt VBDs in households | 3,44 | 3,94 |
| **12** | What are complementary strategies to improve the local detection capacity | 3,39 | 3,83 |
| **37** | What shall we teach a new generation of professionals to prevent or decrease the risk of VBDs | 3,17 | 3,11 |
| **75** | What the local capacities for product development for NTDs | 2,94 | 3,11 |
| **67** | Are the VBDs a priority for the community | 2,78 | 3,39 |
|  | | | |
| Cluster | **Vectors** | **3,26** | **3,01** |
| **5** | What is the value of mosquitoe infection index in the prevision of outbreaks | 3,67 | 3,22 |
| **7** | Are vector capacity and longevity changing/increasing in the urban areas at the moment | 3,67 | 3 |
| **20** | Evaluate the behavior changes of the vector after an intervention | 3,56 | 3 |
| **21** | Assess the health impact of vector control based on epidemiological and entomological metrics | 3,56 | 3,39 |
| **58** | What are the vector density threshold for the disease transmission to occur | 3,33 | 3,44 |
| **34** | Can low vector infestation (house index) cause outbreaks in large urban settings | 2,5 | 2,56 |
| **82** | What are the best patterns to evaluate vector capacity and unexpected species | 2,5 | 2,44 |
|  | | | |
| Cluster | **City responsabity** | **3,2** | **3,48** |
| **13** | What are the sanitation waste management strategies that can help prevent VBDs | 3,83 | 4,22 |
| **40** | How can sustainable urban planning contribute to VBDs control | 3,50 | 4 |
| **65** | Impact of climate change in transmission of VBDs in urban area | 3,44 | 3,28 |
| **93** | How to monitor inner city development to minimize the risk of vector borne diseases introduction | 3,33 | 3,78 |
| **9** | What is the burden of the transmission in urban areas | 3,17 | 3,72 |
| **41** | How the mixed pattern of sanitation within cities can contribute to the maintenance of VBDs circulation | 3,17 | 3,5 |
| **50** | How can climate and health services collaborate better together to prevent VBDs | 3,06 | 3,39 |
| **36** | How can city growth can prevent VBDs | 2,94 | 3,28 |
| **8** | What do we mean about urban areas | 2,33 | 2,11 |
|  | | | |
| Cluster | **Clinics** | **3,16** | **3** |
| **17** | What is the impact of use of RDT on clinical outcomes for patient | 3,72 | 3,56 |
| **24** | Need for more/new parameters to improve our lab test evaluation | 3,56 | 2,78 |
| **85** | What will we do to promote implementation research on RDT | 3,33 | 3,22 |
| **48** | How to ensure quality of RDT available in the market | 3,28 | 3,94 |
| **89** | What are the source of heterogeneity and comparative performance of the available RDT | 3,28 | 2,94 |
| **80** | What diagnostic algorithm work in the routine clinical settings | 3,28 | 3,44 |
| **31** | Identify biomarkers of severity | 3,22 | 2,5 |
| **49** | How many RDT do we need for a single disease | 2,67 | 2,28 |
| **86** | What is the role of multiple testing at the point of care | 2,67 | 2,89 |
| **73** | What is the current state of RDT for leptospirosis | 2,56 | 2,5 |
|  | | | |
| Cluster | **Collaboration** | **3,08** | **3,17** |
| **61** | How can we reduce the time between research implementation/ funding during a public health emergency | 3,5 | 4 |
| **83** | How do we reduce the time lag/delay between research and outbreaks | 3,44 | 3,56 |
| **76** | Should we change the system of incentive to researchers to address health priorities | 2,89 | 3 |
| **77** | Are resources, networking possible in VBDs research | 2,5 | 2,11 |
|  | | | |
| Cluster | **Population mobility** | **2,71** | **2,72** |
| **4** | The influence of population mobility on VBDs | 3,22 | 3,11 |
| **72** | What is the role of the migration of population in the transmission of VBDs | 3,11 | 2,89 |
| **70** | How the mobility of people can be evaluated to perpetuate/sustain | 3 | 2,94 |
| **46** | How can we prevent viremia people to move around | 1,5 | 1,94 |
